# Inadequate dietary energy intake associates with higher prevalence of metabolic syndrome in different groups of hemodialysis patients: a clinical observational study in multiple dialysis centers

**DOI:** 10.1186/s12882-018-1041-z

**Published:** 2018-09-19

**Authors:** Tuyen Van Duong, Te-Chih Wong, Hsi-Hsien Chen, Tzen-Wen Chen, Tso-Hsiao Chen, Yung-Ho Hsu, Sheng-Jeng Peng, Ko-Lin Kuo, Hsiang-Chung Liu, En-Tzu Lin, Chi-Sin Wang, I-Hsin Tseng, Yi-Wei Feng, Tai-Yue Chang, Chien-Tien Su, Shwu-Huey Yang

**Affiliations:** 1 School of Nutrition and Health Sciences, Taipei Medical University, No. 250 Wuxing Street, Taipei, 110 Taiwan; 2 Department of Nutrition and Health Sciences, Chinese Culture University, Taipei, Taiwan; 3 Department of Nephrology, Taipei Medical University Hospital, Taipei, Taiwan; 4 School of Medicine, Taipei Medical University, Taipei, Taiwan; 5 Department of Nephrology, Taipei Medical University- Wan Fang Hospital, Taipei, Taiwan; 6 Division of Nephrology, Department of Internal Medicine, Taipei Medical University- Shuang Ho Hospital, Taipei, Taiwan; 7 Division of Nephrology, Cathay General Hospital, Taipei, Taiwan; 8 Division of Nephrology, Taipei Tzu-Chi Hospital, Taipei, Taiwan; 9 Department of Nephrology, Wei Gong Memorial Hospital, Miaoli, Taiwan; 10 Department of Nephrology, Lotung Poh-Ai Hospital, Yilan, Taiwan; 11 School of Public Health, Taipei Medical University, Taipei, Taiwan; 12 Department of Family Medicine, Taipei Medical University Hospital, Taipei, Taiwan; 13 Research Center of Geriatric Nutrition, Taipei Medical University, Taipei, Taiwan; 14 Nutrition Research Center, Taipei Medical University Hospital, Taipei, Taiwan

**Keywords:** Hemodialysis patients, Inadequate dietary energy intake, Metabolic syndrome, AACE, HMetS

## Abstract

**Background:**

Metabolic syndrome (MetS) has been established as a risk for cardiovascular diseases and mortality in hemodialysis patients. Energy intake (EI) is an important nutritional therapy for preventing MetS. We examined the association of self-reported dietary EI with metabolic abnormalities and MetS among hemodialysis patients.

**Methods:**

A cross-sectional study design was carried out from September 2013 to April 2017 in seven hemodialysis centers. Data were collected from 228 hemodialysis patients with acceptable EI report, 20 years old and above, underwent three hemodialysis sessions a week for at least past 3 months. Dietary EI was evaluated by a three-day dietary record, and confirmed by 24-h dietary recall. Body compositions were measured by bioelectrical impedance analysis. Biochemical data were analyzed using standard laboratory tests. The cut-off values of daily EI were 30 kcal/kg, and 35 kcal/kg for age ≥ 60 years and < 60 years, respectively. MetS was defined by the American Association of Clinical Endocrinologists (AACE-MetS), and Harmonizing Metabolic Syndrome (HMetS). Logistic regression models were utilized for examining the association between EI and MetS. Age, gender, physical activity, hemodialysis vintage, Charlson comorbidity index, high sensitive C-reactive protein, and interdialytic weight gains were adjusted in the multivariate analysis.

**Results:**

The prevalence of inadequate EI, AACE-MetS, and HMetS were 60.5%, 63.2%, and 53.9%, respectively. Inadequate EI was related to higher proportion of metabolic abnormalities and MetS (*p* <  0.05). Results of the multivariate analysis shows that inadequate EI was significantly linked with higher prevalence of impaired fasting glucose (OR = 2.42, *p* <  0.01), overweight/obese (OR = 6.70, *p* <  0.001), elevated waist circumference (OR = 8.17, *p* <  0.001), AACE-MetS (OR = 2.26, *p* <  0.01), and HMetS (OR = 3.52, *p* <  0.01). In subgroup anslysis, inadequate EI strongly associated with AACE-MetS in groups of non-hypertension (OR = 4.09, *p* = 0.004), and non-cardiovascular diseases (OR = 2.59, *p* = 0.012), and with HMetS in all sub-groups of hypertension (OR = 2.59~ 5.33, *p* <  0.05), diabetic group (OR = 8.33, *p* = 0.003), and non-cardiovascular diseases (OR = 3.79, *p* <  0.001).

**Conclusions:**

Inadequate EI and MetS prevalence was high. Energy intake strongly determined MetS in different groups of hemodialysis patients.

## Introduction

The prevalence of treated end-stage renal disease (ESRD) has steadily increased from 2001 to 2014 in all countries, and become a burden to every nation and healthcare system [1]. In 2014, the prevalence of ESRD patients undergoing dialysis in Taiwan was 3093 patients per million population, about 90% of them receiving in-center hemodialysis treatment [1]. It was summarized that nutritional factor was implicated as a risk factor for the development of metabolic in chronic kidney disease, especially in ESRD patients [2].

Nutritional therapy is recognized as an effective approach to prevent metabolic abnormalities and unfavorable outcomes in people with chronic conditions [3–8]. Increased dietary energy intake is mentioned in the National Kidney Foundation-Kidney Disease Outcomes Quality Initiative (K/DOQI) guidelines [9]. It is recommended that consuming enough energy daily guarantees the nitrogen balance and prevents protein catabolism and tissue destruction, which could optimize the nutritional status and hemodialysis outcomes [9]. However, the daily intake of macro-nutrients and micro-nutrients are largely inadequate in hemodialysis patients [10]. More than a half of hemodialysis patients had problems to follow the healthy diet guidelines (related to energy and nutrients intakes) which related behaviors, technical difficulties, physical conditions, time, and food preparation [11]. Inadequate dietary intake is also a possible result of a significant lifestyle change while receiving dialysis treatment. On the other hand, adherence to a complicated and restrictive dietary intake further exacerbates nutrient deficits in this group of patients [9, 12–14].

The prevalence of metabolic syndrome was high in the ESRD patients undergoing hemodialysis [15]. The MetS has been implicated as a risk factor for the development of diabetes, cardiovascular disease, cancer, and all-cause mortality [16–19]. The prevalence of metabolic syndrome varied by different assessment criteria, e.g. 51%, 66.3%, and 75.3% according to National Cholesterol Education Program Adult Treatment Panel III (NCEP ATP III), International Diabetes Federation (IDF), and Harmonizing the Metabolic Syndrome (HMetS) criteria, respectively [20]. This indicated that there was not yet a single definition that could reflect the real spectrum of the epidemiology of MetS. Therefore, in the current study, two definitions were used with different focuses to assess the MetS: The American Association of Clinical Endocrinologists (AACE) definition, focused on hyperglycosemia, was glucocentric [21]; and Harmonizing Metabolic Syndrome definition was agreed by Joint statement from the IDF, American Heart Association (AHA) and the National Heart, Lung, and Blood Institute (NHLBI), the World Heart Federation, the International Atherosclerosis Society, and the International Association for the Study of Obesity, which relayed on collection of abdominal obesity and related CVD risk factors [22].

There were few studies investigated dietary intake among hemodialysis patients. One study compared the dietary intake status between 54 HD patients, and 47 non-HD patients, and between dialysis day and non-dialysis day among elderly people in Brazil [23]. The other study in the United States only examined the association between dietary energy intake and body composition changes in 13 HD patients [24]. In addition, the dietary approach was found as an effective therapy to decrease most of the risks for MetS in a randomized controlled trial [25]. However, hemodialysis patients were with high metabolic syndrome prevalence, and generally have difficulties achieving recommended energy intakes. In our knowledge, the role of dietary energy intake on metabolic disorders among hemodialysis patients remains to be investigated.

This study was to examine the association of inadequate dietary energy intake with metabolic abnormalities and metabolic syndrome among patients who receiving hemodialysis treatment from seven hemodialysis centers. It was hypothesized that hemodialysis patients with reported inadequate dietary energy intake (IDEI) more likely had metabolic abnormalities or metabolic syndrome.

## Methods

### Study design and setting

A cross-sectional study design was carried out from September 2013 to April 2017. We collected data from 492 patients from hemodialysis centers in seven hospitals. The study sample consisted 165 from Taipei Medical University Hospital, 91 from Taipei Medical University – Wan Fang Hospital, 39 from Taipei Medical University – Shuang Ho Hospital, 41 from Cathay General Hospital, 57 from Taipei Tzu-Chi Hospital, 49 from Wei-Gong Memorial Hospital, and 50 from Lotung Poh-Ai Hospital.

### Sample size

**Fig. 1:**
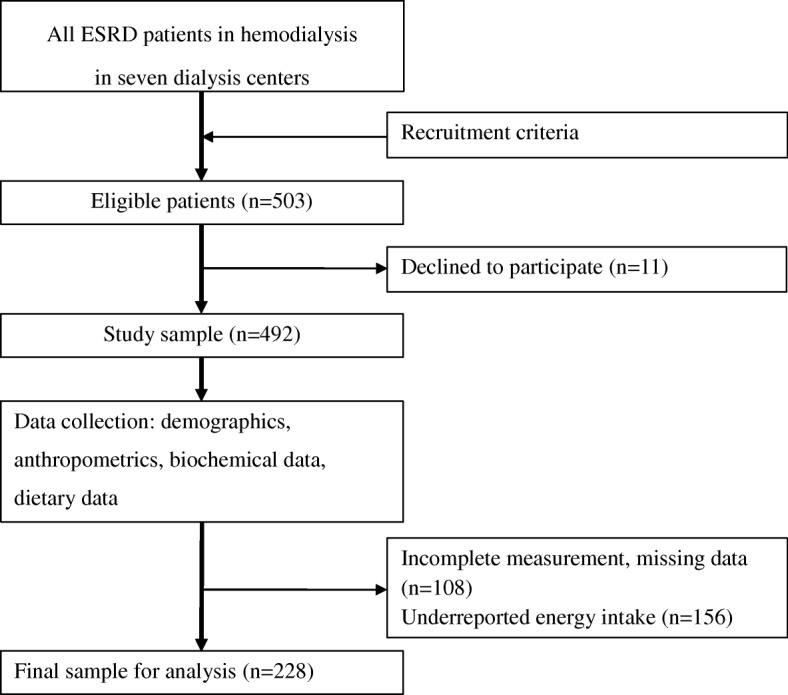
Flow chart of patients sampling and study procedure. ESRD: End-stage renal disease

The sample size in a cross-sectional design is calculated using the formula: $n=\frac{Z^2P\left(1-P\right)}{d^2}$ Where *n* (sample size), *Z* (level of confidence), *P* (expected prevalence), and *d* (precision, corresponding to effect size) [26]. The sample of 92 was calculated with *Z* = 1.96 for type I error of 5%, *P* = 0.745 as the prevalence of MetS was 74.5% in hemodialysis patients [27], and *d* = 0.1 as suggested for a medical study [28]. In the current study, the final sample of 228 patients is adequate for analysis and depicted in Fig. 1.

### Patient recruitment criteria

The study patients in the current study fulfilled the recruitment criteria as mentioned elsewhere [29–31].

### Data collection procedure

The physicians and nurses in each hospital screened for qualified patients who satisfied the recruitment criteria. The interviewers (Registered Dietitians) then contacted the eligible patients and asked for their voluntary participation.

The eligible patients signed the informed consent form before participating in the face-to-face or telephone interviews which conducted by registered dietitians (three-day dietary intake, physical activity). The medical charts were reviewed after the interviews. Anthropometric, and energy expenditure values were also measured. Licensed nurses collected blood samples at the first dialysis session during the study week, biochemical data was then analyzed using available laboratory test kits, the procedure was described in details elsewhere [32].

### Measures

#### Patients’ characteristics

The information regarding age, gender, hemodialysis vintage, comorbidities calculated using the Charlson comorbidity index [33], history of hypertension, cardiovascular diseases, and type 2 diabetes mellitus (T2DM), body mass index, BMI (kg/m^2^), pre-dialysis systolic (SBP) and diastolic (DBP) blood pressure were also assessed using medical records. The waist circumference (WC), body fat mass (FM) were assessed using the bio-electrical impedance analysis device (InBody S10, Biospace, Seoul, Korea), the detailed procedure was described elsewhere [34]. Elevated body fat mass was defined as FM ≥ 25% for men, FM ≥ 30% for women, respectively [35]. Interdialytic weight gains (IDWG) was also calculated. Higher IDWG linked with higher BP in hemodialysis patients [36].

#### Physical activity

The short version of the International Physical Activity Questionnaire was used to evaluate physical activity level. Patients were asked about their time spent (days per week, and minutes per day) on different levels of physical intensity (vigorous, moderate, walking, and sitting), questionnaire took 4 to 15 min to complete [37]. The overall physical activity score was calculated as the sum of minutes spent on activities at different levels of vigorous, moderate, walking, and sitting over last seven days multiplied by 8.0, 4.0, and 3.3, 1.0, respectively [38]. The common method using metabolic equivalent task scored in minute per week (named as MET- min/wk) was used to represent the physical activity [39].

#### Dietary energy intake

We used three-day dietary intake record to assess patient’s intake, and confirmed data by a 24-h dietary recall, the details were mentioned elsewhere [32, 40]. In brief, the information related to names of food, brand, ingredients, cooking methods, portion or weight, meal location and time were collected. The e-Kitchen software, a nutrient analysis software (Nutritionist Edition, Enhancement plus 3, version 2009, Taichung, Taiwan) was used for analyzing nutrients.

The recommended daily dietary energy intake was ≥35 kcal/kg for patients younger than 60 years old, and ≥ 30 kcal/kg for those who 60 years old or older, respectively [9]. Inadequate dietary energy intake was defined as patients consumed less than the recommended levels. In order to enhance the reliability of measures and analysis, the under-reported dietary energy intake (EI) data were excluded from the final analysis if the ratio of EI:REE < 1.27 [41]. The results of the analysis were not affected by excluding the under-reporters in the study [42]. The resting energy expenditure (REE) was assessed using a hand-held indirect calorimeter, named MedGem (Microlife USA, Dunedin, FL). A modified Weir equation together with a fixed respiratory exchange ratio of 0.85 were used to estimate carbon dioxide production. Patients wore a nose clip and a mouthpiece, then breathe normally for about 7–10 min, or until the volume of oxygen is stable. The MedGem has been validated against several metabolic calorimeters such as Douglas Bag method [43], and metabolic cart systems [44, 45]. This device has the similar accuracy of commonly used prediction equations such as the WHO/FAU/UNU, Mifflin, or Harris–Benedict equations [46], and used in hemodialysis patients [47].

#### The biochemical values

Fasting blood glucose (FPG), fasting plasma insulin (FPI), total cholesterol (TC), triglyceride (TG), high-density lipoprotein cholesterol (HDL-C), low-density lipoprotein cholesterol (LDL-C), high sensitive C-reactive protein (hs-CRP), Creatinine, Albumin, intact parathyroid hormone (iPTH), the normalized protein nitrogen appearance (nPNA) was estimated using the formula: nPNA = Pre-BUN/[25.8 + 1.15*(eKt/V) + 56.4/(eKt/V)] + 0.168, where pre-BUN is pre-dialysis blood urea nitrogen (mg/dL), post-BUN is post-dialysis blood urea nitrogen (mg/dl), and equilibrated Kt/V is dialysis quality [48].

#### Diagnosis of metabolic syndrome (MetS)

The MetS was classified by American Association of Clinical Endocrinologists (AACE), hereafter referred as AACE-MetS [21]. Patients were identified as MetS if they had (1) and any of the criteria (2), or (3), or (4). (1) Impaired fasting glucose (IFG) which patients had FPG ≥ 100 mg/dL, or previously diagnosed T2DM [49]. (2) Overweight or obese (BMI ≥ 24.0 kg/m^2^ for Taiwanese) [50]. (3) TG ≥150 mg/dL, HDL-C < 40 mg/dL for men or HDL-C < 50 mg/dL for women. (4) SBP ≥ 130 mmHg or DBP ≥ 85 mmHg.

To affirm the non-spurious association, the Harmonizing Metabolic Syndrome definition (HMetS) was also used to evaluate MetS. Patients were classified as MetS if they have three or more abnormalities (WC ≥ 90 cm for men, WC ≥ 80 cm for women, TG ≥150 mg/dL, low HDL-C, high BP, or IFG) [22].

#### Other biochemical value classifications

The lipid profile (LDL-C ≥ 100 mg/dL, and TC ≥ 200 mg/dL) [51], inflammation maker (high sensitive-CRP > 0.5 mg/dL) [52], elevated insulin (FPI ≥ 12 mU/L) [53, 54], iPTH ≥300 pg/mL [55]. In addition, the poor nutritional status including nPNA < 1.0 g/kg, serum albumin (Alb) ≤ 3.5 mg/dL, and serum creatinine (Cr) ≤ 7.5 mg/dL [56].

### Statistical analysis

The study sample was described using mean ± standard deviation (SD), or median (interquartile range), or frequency (percentage). The continuous variables were tested for normality by using a Shapiro-Wilk’s test [57, 58], and histograms, box plots, and normal Q-Q plots were examined. The ANOVA, Mann-Whitney U test, or Chi-Square test were recruited in order to compare characteristics and metabolic parameters of the adequate and inadequate EI groups. The bivariate logistic regression models were recruited for examining associations of patients’ characteristics, dietary intake with metabolic abnormalities and MetS. The multivariate logistic regression analyses were then utilized for examining the association of inadequate dietary intake of nutrients with metabolic abnormalities and MetS. The sub-group analyses were performed in different groups of diabetes mellitus, hypertension, and cardiovascular diseases. Patients’ gender, age, physical activity, hemodialysis vintage, Charlson comorbidity index (CCI), hs-CRP, and IDWG were controlled in the multivariate analyses as they showed the associations with metabolic syndrome [59–63]. The analyses were performed for both diagnosed criteria of MetS (AACE-MetS and H-MetS) to affirm the non-spurious association. The IBM SPSS software version 20.0 for Windows (IBM Corp., New York, USA) was used for all analyses. The statistically significant level was set at *P* value < 0.05.

## Results

The mean ± SD of age, hemodialysis vintage, physical activity, CCI, and interdialytic weight gains were 59.4 ± 11.3, 5.5 ± 5.0, 4831.3 ± 1893.1, 4.6 ± 1.5, and 3.0 ± 1.7, respectively. Of study sample, there were 64.9% men, 38.2% diabetes, 48.2% hypertension, and 29.8% cardiovascular diseases, 28.5% with an elevated level of hs-CRP, 54.5% elevated body fat mass. The REE was lower in patients with inadequate EI (1014.5 ± 280.4) than those with adequate EI (1100.9 ± 274.7), with *p* = 0.023. Regarding metabolic abnormalities, the prevalence of IFG, overweight or obese, elevated WC, high BP, high TG, and low HDL-C were 64.9%, 36.4%, 26.3%, 81.6%, 39.0%, and 61.0%, respectively. The prevalence of metabolic syndrome was 63.2% as diagnosed by AACE criteria, and 53.9% as diagnosed by HMetS criteria. The prevalence of the metabolic abnormalities (not hypertension) and syndromes were statistically significantly higher in hemodialysis patients with inadequate EI than those who with adequate EI (Table 1). Out of patients, 60.5% reported less than the recommendation level of dietary energy intake. Patients with inadequate EI more likely consumed inadequate protein and fat, but consumed less mineral, water, and vitamin than those with adequate EI (Table 2).

**Table 1:** Characteristics, and metabolic parameters, and other biochemical values in hemodialysis patients^a^

Variables	Total sample (*n* = 228)	Adequate EI (*n* = 90)	Inadequate EI (*n* = 138) ^b^	*P* value ^c^
Characteristics				
Age, years	59.4 ± 11.3	59.9 ± 10.8	59.1 ± 11.6	0.630
Gender, male	148 (64.9)	57 (63.3)	91 (65.9)	0.687
Hemodialysis vintage, years	5.5 ± 5.0	6.9 ± 5.9	4.5 ± 4.0	< 0.001
CCI	4.6 ± 1.5	4.7 ± 1.5	4.5 ± 1.6	0.327
Diabetes mellitus	87 (38.2)	24 (26.7)	63 (45.7)	0.004
Hypertension	110 (48.2)	45 (50.0)	65 (47.1)	0.669
Cardiovascular diseases	68 (29.8)	26 (28.9)	42 (30.4)	0.803
Physical activity, MET score	4831.3 ± 1893.1	4984.9 ± 2033.2	4732.6 ± 1798.1	0.330
Height, cm	162.4 ± 8.3	161.4 ± 7.0	163.0 ± 9.0	0.149
Weight, kg	61.4 ± 12.3	55.1 ± 8.9	65.4 ± 12.6	0.000
IDWG, %	3.0 ± 1.7	2.9 ± 2.0	3.1 ± 1.5	0.227
FM, %	27.2 ± 10.0	23.4 ± 9.1	29.7 ± 9.9	< 0.001
Elevated FM	122 (54.5)	34 (38.2)	88 (65.2)	< 0.001
REE, kcal/day	1048.6 ± 280.8	1100.9 ± 274.7	1014.5 ± 280.4	0.023
Metabolic abnormalities				
FPG	105.3 (90.5, 145.2)	97.3 (90.3, 134.0)	114.0 (93.6, 153.8)	0.025
IFG	148 (64.9)	47 (52.2)	101 (73.2)	0.001
BMI, kg/m^2^	23.2 ± 3.8	21.1 ± 2.6	24.5 ± 3.9	< 0.001
BMI ≥ 24 (kg/m^2^)	83 (36.4)	13 (14.4)	70 (50.7)	< 0.001
WC, cm	81.1 ± 10.4	75.7 ± 7.5	87.6 ± 36.4	0.002
Elevated WC	60 (26.3)	8 (8.9)	52 (37.7)	< 0.001
TG, mg/dL	115.0 (82.9, 202.6)	99.1 (78.0, 155.4)	136.8 (85.0, 250.5)	0.004
High TG ≥ 150 (mg/dL)	89 (39.0)	27 (30.0)	62 (44.9)	0.024
HDL-C, mg/dL	41.6 ± 22.1	45.8 ± 21.0	38.9 ± 22.4	0.021
Low HDL-C	139 (61.0)	47 (52.2)	92 (66.7)	0.029
SBP, mmHg	146.5 ± 22.7	149.5 ± 24.0	144.3 ± 21.3	0.089
DBP, mmHg	80.0 ± 18.2	79.8 ± 19.0	79.9 ± 17.6	0.959
High BP	186 (81.6)	73 (81.1)	113 (81.9)	0.883
AACE-MetS ^d^	144 (63.2)	46 (51.1)	98 (71.0)	0.002
HMetS^e^	123 (53.9)	33 (36.7)	90 (65.2)	< 0.001
Other biochemical values				
TC, mg/dL	168.3 ± 37.9	163.8 ± 33.7	170.7 ± 40.0	0.178
TC ≥ 200 mg/dL	39 (17.1)	10 (11.1)	29 (21.0)	0.052
LDL-C, mg/dL	102.1 ± 32.5	98.0 ± 31.0	104.6 ± 32.9	0.130
LDL-C ≥ 100 mg/dL	41 (18.0)	13 (14.4)	28 (20.3)	0.261
FPI, μU/mL	15.2 (7.9, 31.9)	12.7 (6.8, 26.5)	18.6 (9.3, 35.7)	0.004
FPI ≥ 12 μU/mL	142 (62.3)	47 (52.2)	95 (68.8)	0.011
hs-CRP, mg/dL	0.3 (0.1, 0.6)	0.2 (0.1, 0.5)	0.3 (0.1, 0.6)	0.277
hs-CRP ≥ 0.5 mg/dL	65 (28.5)	23 (25.6)	42 (30.4)	0.425
iPTH, pg/mL	225.2 (80.6, 409.1)	231.0 (68.5, 441.2)	223.9 (94.4, 382.7)	0.916
iPTH ≥300 pg/mL	93 (40.8)	38 (42.2)	55 (39.9)	0.722
Creatinine, mg/dL	11.1 ± 1.9	10.8 ± 1.7	11.3 ± 2.1	0.077
Creatinine ≤7.5 mg/dL	8 (3.5)	6 (6.7)	2 (1.4)	0.036
Albumin, mg/dL	4.0 ± 0.4	4.0 ± 0.4	4.0 ± 0.4	0.992
Albumin ≤3.5 mg/dL	24 (10.5)	8 (8.9)	16 (11.6)	0.515
Pre-BUN, mg/dL	72.9 ± 20.9	76.7 ± 21.2	70.2 ± 20.7	0.023
Post-BUN, mg/dL	19.9 ± 7.8	19.0 ± 7.6	20.7 ± 7.9	0.106
eKt/V	1.6 ± 0.3	1.8 ± 0.4	1.5 ± 0.3	< 0.001
nPNA, g/kg	1.4 ± 0.4	1.4 ± 0.4	1.3 ± 0.4	< 0.001
nPNA < 1.0 g/kg	29 (12.7)	7 (7.8)	22 (15.9)	0.071

CCI: Charlson comorbidity index, MET: metabolic equivalent minute/week, IDWG, interdialytic weight gains, FM: fat mass, IFG: Impaired fasting glucose, BMI: body mass index, WC: waist circumference, TG: triglyceride, HDL-C: high-density lipoprotein cholesterol, BP: blood pressure, SBP: systolic blood pressure, DBP: diastolic blood pressure, TC: total cholesterol, LDL-C: low-density lipoprotein cholesterol, FPI: fasting plasma insulin, hs-CRP: high sensitive C-reactive protein, iPTH, intact parathyroid hormone, nPNA = normalized protein nitrogen appearance

^a^Categorical data is shown as n (%). Continuous data is presented as mean ± SD, or median (interquartile range)

^b^Inadequate energy intake was classified as EI < 30 kcal/kg/day for age 60 and above; < 35 for age less than 60

^c^Independent-samples T-test, Mann-Whitney U test, or Chi-square tests are performed

^d^Metabolic syndrome diagnosed by American Association of Clinical Endocrinologists (IFG plus any other abnormality: overweight/obese, high TG, low HDL, high blood pressure)

^e^Metabolic syndrome diagnosed by Harmonizing Metabolic Syndrome (three or more abnormalities: Elevated WC, IFG, low HDL, high TG, high blood pressure)

**Table 2:** Dietary intake among hemodialysis patients^a^

Daily dietary intake ^b^	Total sample (*n* = 228)	Adequate EI (*n* = 90)	Inadequate EI (*n* = 138) ^c^	*P* value ^d^
Macronutrients				
Energy intake, kcal	1885.0 ± 477.2	2182.6 ± 448.9	1690.9 ± 387.7	< 0.001
Energy intake, kcal/kg	31.5 ± 8.8	39.8 ± 7.0	26.1 ± 4.6	< 0.001
Protein, g/kg IBW	1.2 ± 0.3	1.4 ± 0.3	1.1 ± 0.3	< 0.001
Protein < 1.2 g/kg IBW	132 (57.9)	28 (31.1)	104 (75.4)	< 0.001
Protein, (%EI)	15.0 ± 3.0	14.6 ± 2.7	15.2 ± 3.2	0.090
Protein < 15% EI	118 (51.8)	52 (57.8)	66 (47.8)	0.142
Carbohydrate, g	222.1 ± 68.8	252.9 ± 71.5	202.0 ± 59.1	< 0.001
Carbohydrate, (%EI)	47.6 ± 8.6	46.5 ± 8.2	48.3 ± 8.9	0.138
Carbohydrate < 45%EI	80 (35.1)	33 (36.7)	47 (34.1)	0.687
Total fat, g	78.3 ± 27.0	92.5 ± 26.5	69.0 ± 23.0	< 0.001
Total fat, (%EI)	37.1 ± 7.8	38.2 ± 7.4	36.4 ± 8.1	0.100
SFA (%EI)	13.4 (8.0, 69.4)	10.6 (8.0, 62.9)	37.9 (8.5, 73.7)	0.083
SFA ≥10% EI	143 (62.7)	53 (58.9)	90 (65.2)	0.334
MUFA (%EI)	18.0 (10.6, 76.0)	13.4 (9.8, 73.4)	41.8 (11.3, 80.2)	0.024
MUFA ≥20% EI	109 (47.8)	34 (37.8)	75 (54.3)	0.014
PUFA (%EI)	17.6 (8.7, 60.6)	12.2 (7.6, 52.0)	32.8 (9.6, 62.9)	0.015
PUFA ≥10% EI	155 (68.0)	55 (61.1)	100 (72.5)	0.072
SFA/UFA ratio	0.5 ± 0.2	0.5 ± 0.2	0.5 ± 0.2	0.869
UFA/SFA ratio	2.3 ± 0.7	2.3 ± 0.6	2.3 ± 0.8	0.426
Micronutrients				
Mineral and Water				
Sodium, mg/d	1254.8 ± 897.6	1576.9 ± 1108.9	1044.6 ± 650.7	< 0.001
Sodium > 1800 mg/d	43 (18.9)	29 (32.2)	14 (10.1)	< 0.001
Fluid, mL/d	1382.6 ± 480.5	1493.7 ± 506.7	1310.2 ± 449.8	0.005
Fluid > 1500 mL/d	78 (34.2)	38 (42.2)	40 (29.0)	0.039
Potassium, mg/d	1445.2 ± 582.6	1616.3 ± 575.7	1333.6 ± 561.5	< 0.001
Phosphate, mg/d	694.9 ± 257.9	799.7 ± 270.2	626.6 ± 225.6	< 0.001
Calcium, mg/d	291.3 ± 177.2	336.9 ± 190.7	261.6 ± 161.6	0.002
Iron, mg/d	8.6 ± 4.6	9.7 ± 5.3	7.8 ± 4.0	0.003
Zinc, mg/d	8.1 ± 3.8	9.3 ± 4.0	7.3 ± 3.5	< 0.001
Vitamins				
Vitamin B1 (thiamin), mg/d	0.8 ± 0.6	1.0 ± 0.6	0.8 ± 0.6	0.008
Vitamin B2 (riboflavin), mg/d	0.9 ± 0.5	1.0 ± 0.6	0.8 ± 0.5	0.001
Niacin (B3), mg/d	11.8 ± 6.3	13.9 ± 7.0	10.5 ± 5.5	< 0.001
Vitamin B6 (pyridoxine), mg/d	1.2 ± 0.9	1.4 ± 1.0	1.0 ± 0.7	0.015
Vitamin B12, μg/d	3.8 ± 3.7	4.5 ± 4.1	3.4 ± 3.4	0.022
Vitamin C, mg/d	90.6 ± 63.5	95.6 ± 59.7	87.3 ± 65.8	0.335
Vitamin E, mg/d ^†^	12.6 ± 10.3	12.9 ± 11.1	12.6 ± 10.3	0.717

EI: energy intake, IBW: ideal body weight, SFA: saturated fatty acid, MUFA: mono-unsaturated fatty acid, PUFA: poly-unsaturated fatty acid, UFA: unsaturated fatty acid

^a^Categorical data is shown as n (%). Continuous data is presented as mean ± SD, or median (interquartile range)

^b^Target values recommended by Standing Committee on the Scientific Evaluation of Dietary Reference Intakes, Food and Nutrition Board, Institute of Medicine; the European Best Practice Guideline on Nutrition and Chronic Kidney Disease; and Clinical Practice Guidelines for Nutrition in Chronic Renal Failure

^c^Inadequate energy intake was classified as EI < 30 kcal/kg/day for age 60 and above; < 35 for age less than 60

^d^Independent-samples T-test, Mann-Whitney U test, or Chi-square tests are performed

The results of bivariate logistic regression analyses presented that higher age associated with higher prevalence of IFG and AACE-MetS with odd ratio, OR = 1.03, 95% confidence interval, 95%CI, 1.00–1.05, *p* <  0.05, and OR = 1.03, 95%CI, 1.01–1.06, *p* <  0.05, respectively. Men experienced higher prevalence of overweight or obesity (OR = 1.85, 95%CI, 1.03–3.33, *p* <  0.05), but lower prevalence of elevated waist circumference (OR = 0.32, 95%CI, 0.17–0.59, *p* <  0.001) than women. Hemodialysis vintage was negatively associated with IFG (OR = 0.91, 95%CI, 0.86–0.97, *p* <  0.001), Overweight/obese (OR = 0.92, 95%CI, 0.86–0.98, *p* <  0.05), high TG (OR = 0.94, 95%CI, 0.89–0.99, *p* <  0.05), low HDL-C (OR = 0.95, 95%CI, 0.90–0.99, *p* <  0.05), AACE-MetS (OR = 0.90, 95%CI, 0.85–0.96, *p* <  0.001), and HMetS (OR = 0.94, 95%CI, 0.89–0.99, *p* <  0.05), respectively. Charlson comorbidity index was positively associated with IFG (OR = 1.38, 95%CI, 1.14–1.67, *p* <  0.001), Overweight/obese (OR = 1.21, 95%CI, 1.01–1.45, *p* <  0.05), AACE-MetS (OR = 1.44, 95%CI, 1.19–1.74, *p* <  0.001), and HMetS (OR = 1.28, 95%CI, 1.07–1.53, *p* <  0.01), respectively. Interdialytic weight gains was positively linked with IFG (OR = 1.21, 95%CI, 1.03–1.43, *p* <  0.05), AACE-MetS (OR = 1.22, 95%CI, 1.04–1.43, *p* <  0.05), and HMetS (OR = 1.24, 95%CI, 1.06–1.46, *p* <  0.01), respectively (Table 3).

**Table 3:** Bivariate analysis the effects of personal factors and dietary intake on metabolic abnormalities and metabolic syndrome

	Metabolic abnormalities						AACE-MetS ^a^	HMetS ^b^
	IFG	Overweight/Obese	Elevated WC	High TG	Low HDL-C	High BP		
	OR (95% CI)	OR (95% CI)	OR (95% CI)	OR (95% CI)	OR (95% CI)	OR (95% CI)	OR (95% CI)	OR (95% CI)
Age, years	1.03 (1.00, 1.05)^*^	1.01 (0.99, 1.04)	1.00 (0.98, 1.03)	1.00 (0.98, 1.03)	1.01 (0.98, 1.03)	1.00 (0.97, 1.03)	1.03 (1.01, 1.06)^*^	1.01 (0.98, 1.03)
Male gender	1.18 (0.67, 2.07)	1.85 (1.03, 3.33)^*^	0.32 (0.17, 0.59)^***^	1.20 (0.68, 2.10)	1.25 (0.72, 2.18)	1.69 (0.86, 3.34)	1.13 (0.65, 1.99)	1.18 (0.69, 2.04)
Hemodialysis vintage, years	0.91 (0.86, 0.97)^***^	0.92 (0.86, 0.98)^*^	0.97 (0.91, 1.03)	0.94 (0.89, 0.99)^*^	0.95 (0.90, 0.99)^*^	1.01 (0.95, 1.09)	0.90 (0.85, 0.96)^***^	0.94 (0.89, 0.99)^*^
CCI	1.38 (1.14, 1.67)^***^	1.21 (1.01, 1.45)^*^	1.07 (0.88, 1.29)	1.11 (0.94, 1.33)	1.14 (0.95, 1.36)	1.12 (0.89, 1.39)	1.44 (1.19, 1.74)^***^	1.28 (1.07, 1.53)^**^
Physical activity, (10% MET increment)	1.07 (0.97, 1.18)	0.99 (0.90, 1.08)	0.92 (0.83, 1.02)	0.96 (0.87, 1.05)	0.93 (0.85, 1.03)	1.07 (0.95, 1.20)	1.06 (0.97, 1.17)	0.99 (0.91, 1.09)
hs-CRP > 0.5 mg/dL	1.45 (0.78, 2.70)	1.63 (0.90, 2.93)	1.37 (0.72, 2.58)	1.16 (0.64, 2.08)	1.36 (0.75, 2.49)	0.66 (0.33, 1.35)	1.20 (0.66, 2.19)	1.41 (0.79, 2.53)
IDWG, %	1.21 (1.03, 1.43)^*^	1.00 (0.85, 1.16)	1.10 (0.93, 1.31)	1.12 (0.96, 1.32)	1.13 (0.97, 1.32)	1.05 (0.87, 1.28)	1.22 (1.04, 1.43)^*^	1.24 (1.06, 1.46)^**^
Dietary intake								
Inadequate EI	2.50 (1.43, 4.37)^***^	6.10 (3.10, 11.99)^***^	6.20 (2.78, 13.84)^***^	1.90 (1.09, 3.34)^*^	1.83 (1.06, 3.15)^*^	1.05 (0.53, 2.08)	2.34 (1.35, 4.08)^**^	3.24 (1.86, 5.63)^***^
Protein < 1.2 g/kg IBW	1.20 (0.69, 2.08)	0.85 (0.50, 1.47)	0.93 (0.52, 1.70)	1.12 (0.65, 1.92)	1.31 (0.76, 2.23)	0.92 (0.47, 1.82)	1.05 (0.61, 1.81)	1.32 (0.78, 2.23)
Carbohydrate < 45%EI	1.42 (0.79, 2.54)	1.08 (0.61, 1.89)	1.95 (1.07, 3.57)^*^	0.83 (0.48, 1.46)	0.87 (0.50, 1.51)	1.44 (0.69, 3.00)	1.46 (0.82, 2.59)	1.25 (0.72, 2.16)
SFA ≥10% EI	1.41 (0.81, 2.46)	0.85 (0.49, 1.48)	0.64 (0.35, 1.17)	0.94 (0.54, 1.62)	0.91 (0.53, 1.58)	1.91 (0.97, 3.75)	1.45 (0.84, 2.53)	0.92 (0.54, 1.57)
MUFA ≥20% EI	2.25 (1.28, 3.94)^**^	1.11 (0.64, 1.90)	0.71 (0.39, 1.30)	0.89 (0.52, 1.52)	1.12 (0.66, 1.91)	1.44 (0.73, 2.84)	2.19 (1.26, 3.81)^**^	1.17 (0.69, 1.97)
PUFA ≥10% EI	1.04 (0.58, 1.85)	0.96 (0.54, 1.72)	1.02 (0.54, 1.93)	1.14 (0.64, 2.02)	1.14 (0.64, 2.00)	1.58 (0.79, 3.15)	1.10 (0.62, 1.95)	1.12 (0.64, 1.95)
SFA/UFA ratio	0.67 (0.13, 3.43)	0.30 (0.05, 1.80)	0.33 (0.05, 2.41)	0.49 (0.09, 2.64)	2.23 (0.41, 12.15)	2.50 (0.27, 23.46)	0.86 (0.17, 4.42)	0.53 (0.11, 2.62)
UFA/SFA ratio	1.11 (0.75, 1.63)	1.37 (0.93, 2.01)	1.51 (1.00, 2.28)	1.28 (0.88, 1.86)	1.08 (0.74, 1.58)	0.81 (0.51, 1.29)	1.05 (0.72, 1.55)	1.46 (0.99, 2.14)
Sodium > 1800 mg	0.62 (0.32, 1.22)	1.04 (0.53, 2.08)	0.82 (0.38, 1.78)	1.16 (0.59, 2.27)	1.61 (0.79, 3.29)	0.99 (0.42, 2.31)	0.68 (0.35, 1.34)	0.98 (0.50, 1.90)
Fluid > 1500 mL	0.68 (0.38, 1.19)	1.73 (0.99, 3.04)	1.55 (0.84, 2.85)	1.34 (0.76, 2.33)	1.45 (0.82, 2.57)	0.92 (0.46, 1.86)	0.70 (0.40, 1.23)	1.16 (0.67, 2.02)

IFG: Impaired fasting glucose, WC: waist circumference, TG: triglyceride, HDL-C: high-density lipoprotein cholesterol, BP: blood pressure, CCI: Charlson comorbidity index, MET: metabolic equivalent minute/week, hs-CRP: high sensitive C-reactive protein, IDWG, interdialytic weight gains; BF: body fat; EI: energy intake, IBW: ideal body weight, SFA: saturated fatty acid, MUFA: mono-unsaturated fatty acid, PUFA: polyunsaturated fatty acid, UFA: unsaturated fatty acid

^a^Metabolic syndrome diagnosed by American Association of Clinical Endocrinologists (IFG plus any other abnormality: overweight/obese, high TG, low HDL-C, high BP)

^b^Metabolic syndrome diagnosed by Harmonizing Metabolic Syndrome (three or more abnormalities: elevated WC, IFG, low HDL-C, high TG, high BP)

Significant level at ^*^ *p* < 0.05, ^**^ *p* < 0.01, ^***^ *p* < 0.001

Reported inadequate dietary energy intake associated with 1.83–6.20 folds of metabolic abnormalities or metabolic syndrome. It was significantly linked to higher prevalence of IFG (OR = 2.50, 95%CI, 1.43–4.37, *p* <  0.001), overweight/obese (OR = 6.10, 95%CI, 3.10–11.99, *p* <  0.001), elevated waist circumference (OR = 6.20, 2.78–13.84, *p* <  0.001), high triglyceride (OR = 1.90, 95%CI, 1.09–3.34, *p* <  0.05), low HDL-C (OR = 1.83, 95%CI, 1.06–3.15, *p* <  0.05), AACE -MetS (OR = 2.34, 95%CI, 1.35–4.06, *p* <  0.01), and HMetS (OR = 3.24, 95%CI, 1.86–5.63, *p* <  0.001), respectively. The sodium and fluid intake were not associated with metabolic abnormalities or MetS (Table 3).

The associations of inadequate energy intake with metabolic abnormalities, AACE-MetS, and HMetS were stronger by 2.26 to 8.17 folds after adjusted for gender, age, physical activity, hemodialysis vintage, Charlson comorbidity index (CCI), hs-CRP, and IDWG in multivariate analyses. Inadequate energy intake did not show the significant association with high TG, low HDL-C or high blood pressure (Table 4). On the other hand, the consumption of MUFA greater or equal to 20% of EI is associated with higher likelihood of having IFG (OR = 2.85, 95%CI, 1.39–5.87, *p* <  0.01), and AACE-MetS (OR = 3.01, 95%CI, 1.45–6.26, *p* <  0.01, Table 4).

**Table 4:** Associations of dietary intake and metabolic abnormalities and metabolic syndrome via multivariate logistic regression analyses ^a^

Dietary intake	Metabolic abnormalities						AACE-MetS ^b^	HMetS ^c^
	IFG	Overweight/Obese	Elevated WC	High TG	Low HDL-C	High BP		
	OR (95% CI)	OR (95% CI)	OR (95% CI)	OR (95% CI)	OR (95% CI)	OR (95% CI)	OR (95% CI)	OR (95% CI)
Inadequate EI	2.42 (1.30, 4.51)^**^	6.70 (3.25, 13.81)^***^	8.17 (3.33, 20.01)^***^	1.72 (0.95, 3.10)	1.67 (0.94, 2.98)	1.18 (0.57, 2.43)	2.26 (1.21, 4.23)^*^	3.52 (1.91, 6.50)^**^
Protein < 1.2 g/kg IBW	0.94 (0.51, 1.74)	0.74 (0.41, 1.33)	0.81 (0.42, 1.56)	0.95 (0.54, 1.69)	1.14 (0.64, 2.02)	0.99 (0.48, 2.04)	0.78 (0.42, 1.46)	1.15 (0.64, 2.04)
Carbohydrate < 45%EI	1.30 (0.69, 2.46)	1.12 (0.61, 2.05)	1.88 (0.97, 3.66)	0.86 (0.48, 1.56)	0.90 (0.50, 1.62)	1.57 (0.72, 3.43)	1.30 (0.69, 2.45)	1.33 (0.73, 2.41)
SFA ≥10% EI	1.70 (0.87, 3.31)	0.94 (0.50, 1.77)	0.90 (0.45, 1.80)	1.22 (0.66, 2.28)	1.22 (0.66, 2.29)	2.01 (0.93, 4.32)	1.88 (0.96, 3.70)	1.25 (0.66, 2.34)
MUFA ≥20% EI	2.85 (1.39, 5.87)^**^	1.20 (0.63, 2.30)	1.09 (0.52, 2.26)	1.07 (0.56, 2.03)	1.59 (0.83, 3.04)	1.40 (0.62, 3.16)	3.01 (1.45, 6.26)^**^	1.55 (0.81, 2.99)
PUFA ≥10% EI	0.99 (0.52, 1.90)	1.07 (0.58, 1.98)	1.27 (0.64, 2.51)	1.30 (0.71, 2.38)	1.33 (0.73, 2.44)	1.60 (0.77, 3.32)	1.09 (0.57, 2.09)	1.32 (0.72, 2.45)
SFA/UFA ratio	1.03 (0.17, 6.15)	0.47 (0.07, 3.05)	0.46 (0.06, 3.46)	0.82 (0.14, 4.71)	5.13 (0.79, 33.43)	3.07 (0.31, 30.62)	1.54 (0.26, 9.37)	1.12 (0.20, 6.24)
UFA/SFA ratio	0.98 (0.63, 1.52)	1.24 (0.82, 1.88)	1.34 (0.86, 2.10)	1.11 (0.74, 1.66)	0.89 (0.59, 1.35)	0.80 (0.49, 1.31)	0.90 (0.58, 1.39)	1.24 (0.81, 1.89)
Sodium > 1800 mg	0.63 (0.30, 1.31)	0.96 (0.47, 1.97)	0.96 (0.42, 2.20)	1.16 (0.57, 2.34)	1.66 (0.79, 3.51)	0.95 (0.39, 2.31)	0.73 (0.35, 1.52)	0.99 (0.49, 2.01)
Fluid > 1500 mL	0.60 (0.32, 1.15)	1.68 (0.91, 3.10)	1.76 (0.89, 3.46)	1.22 (0.67, 2.23)	1.30 (0.70, 2.40)	0.79 (0.37, 1.66)	0.61 (0.32, 1.17)	0.97 (0.52, 1.78)

IFG: Impaired fasting glucose, WC: waist circumference, TG: triglyceride, HDL-C: high-density lipoprotein cholesterol, BP: blood pressure, EI: energy intake, IBW: ideal body weight, SFA: saturated fatty acid, MUFA: mono-unsaturated fatty acid, PUFA: polyunsaturated fatty acid, UFA: unsaturated fatty acid

^a^The analysis was adjusted for age, gender, hemodialysis vintage, Charlson comorbidity index, physical activity, high sensitive C-reactive protein, and interdialytic weight gains

^b^Metabolic syndrome diagnosed by American Association of Clinical Endocrinologists (IFG plus any other abnormality: overweight/obese, high TG, low HDL-C, high BP)

^c^Metabolic syndrome diagnosed by Harmonizing Metabolic Syndrome (three or more abnormalities: elevated WC, IFG, low HDL-C, high TG, high BP)

Significant level at ^*^ *p* < 0.05, ^**^ *p* < 0.01, ^***^ *p* < 0.001

In sub-group analyses, inadequate EI showed an significant association with higher prevalence of AACE-MetS in non-hypertension group (OR = 4.09, 95%CI, 1.55–10.77, *p* = 0.004), and non-cardiovascular disease group (OR = 2.59, 95%CI, 1.23–5.42, *p* = 0.012); and associated with HMetS in group of diabetes (OR = 8.33, 95%CI, 2.08–33.37, *p* = 0.003), non-hypertension (OR = 5.33, 95%CI, 1.97–14.40, *p* = 0.001), hypertension (OR = 2.59, 95%CI, 1.05–6.37, *p* = 0.038), and non-CVD (OR = 3.79, 95%CI, 1.80–7.97, *p* < 0.001, Table 5).

**Table 5:** Association between inadequate energy intake and metabolic syndrome in subgroups of medical history^a^

	Inadequate EI		AACE-MetS ^b^			HMetS ^c^	
	(*n* = 138)	*n*	OR (95% CI)	*p*	*n*	OR (95% CI)	*p*
Non-DM (*n* = 141)	75	35	1.15 (0.54, 2.47)	0.718	35	1.91 (0.88, 4.15)	0.101
DM (*n* = 87)	63	63	N/A		55	8.33 (2.08, 33.37)	0.003
Non-HTN (*n* = 118)	73	51	4.09 (1.55, 10.77)	0.004	44	5.33 (1.97, 14.40)	0.001
HTN (*n* = 110)	65	47	1.33 (0.51, 3.51)	0.560	46	2.59 (1.05, 6.37)	0.038
Non-CVD (*n* = 160)	96	68	2.59 (1.23, 5.42)	0.012	62	3.79 (1.80, 7.97)	< 0.001
CVD (*n* = 68)	42	30	1.48 (0.33, 6.75)	0.612	28	3.64 (0.99, 13.36)	0.052

EI: energy intake, DM: diabetes mellitus, HTN: hypertension, CVD: cardiovascular diseases

^a^The analysis was adjusted for age, gender, hemodialysis vintage, Charlson comorbidity index, physical activity, high sensitive C-reactive protein, and interdialytic weight gains

^b^Metabolic syndrome diagnosed by American Association of Clinical Endocrinologists (IFG plus any other abnormality: overweight/obese, high TG, low HDL-C, high BP)

^c^Metabolic syndrome diagnosed by Harmonizing Metabolic Syndrome (three or more abnormalities: elevated WC, IFG, low HDL-C, high TG, high BP)

## Discussion

In the present study, results elucidated that reported inadequate dietary energy intake (IDEI) associated with more MetS abnormalities, and a higher proportion of MetS. The reported IDEI strongly determined 2.26 to 8.17 folds of metabolic abnormalities and MetS diagnosed either by AACE or HMetS criteria. In hemodialysis patients, IDEI disrupts the energy balance, and the nitrogen balance, increases the tissue destruction, and protein catabolism which cause the MetS and exacerbate the dialysis outcomes [64]. On the other hand, the MetS was found to be a high-risk for many chronic health problems such as obesity, T2DM, cardiovascular diseases, cancer, and all-cause of death [16–19]. Therefore, the early MetS identification and nutritional therapy were highly recommended to reduce above adverse health problems [25, 65]. In addition, patients who consumed adequate energy-rich-protein can improve the balance of body protein, body composition which further improve hemodialysis outcomes [66].

The current study showed that about 60% of hemodialysis patients consumed low dietary energy intake. This was in the line with a reliable previous publication in which patients had at most 75% of the energy and protein intake as recommended by K/DOQI guidelines [9]. MetS prevalence was high in the present study (63.2% AACE-MetS, 53.9% HMetS), and in previous studies in Southern Taiwan was 61.0% measured by NCEP-ATP III criteria [15]. In comparison with previous studies, the prevalence of MetS was lower in the current study than that in a study in Brazil (74.5%) using the HMetS criteria [27], and in United States (69.3%) using the NCEP-ATP III criteria [67].

The consumption of PUFA and SFA did not show the significant association with MetS and its abnormalities, while the consumption of MUFA equal or greater than 20% demonstrated the association with higher IFG and AACE-MetS in the current study. In a previous study, no association between PUFA, or SFA, and MetS was found [68]. Inconsistently, a number of previous studies suggested that the consumption of dietary MUFA improves insulin sensitivity. In addition, MUFA intake as a substitution for SFA demonstrated the benefit for reducing the metabolic syndrome [69, 70]. The discrepancy between the findings of this study and other studies could be explained by the cross-sectional design of the current study, the causal relationship is not generated. In addition, the 24-h dietary recall is subject to reporting bias from patients. In practical application, the MUFA was with high density in the Mediterranean dietary pattern (MDP). Strong evidence from several studies and trials proved that the MDP was inversely associated with the incidence of MetS, cardiovascular diseases [71–74]. Therefore, this MDP can be still encouraged and adopted in various population and cultures, with cost-effective serve for preventing the MetS and its components [75]. However, the application is with precaution and more studies are suggested to intensively investigate the MDP effect on the MetS in hemodialysis patients.

The current results illustrated that the inadequate dietary EI was associated with high prevalence of HMetS in different sub-groups. In a study conducted in Italy, the authors found that patients with MetS reported lower energy intake than those without MetS [76]. This suggested that MetS diagnosed by Harmonizing Metabolic Syndrome criteria is more sensitive than AACE-MetS in relation to energy intake. In practice, in order to improve the hemodialysis outcomes, the adequate dietary EI is recommended by the K/DOQI guidelines which can reduce the risks of MetS [9].

The present study demonstrated that the higher prevalence of IFG and AACE-MetS was observed in older patients. The association was also found in previous studies on the general population in Norway, which MetS was diagnosed by either NCEP- ATP III, or IDF criteria [60], and in individuals in the United States [61]. This emphasized that the old people are more likely to have metabolic abnormalities, risks for CVD, and type 2 diabetes. Therefore, the MetS definitions should be specifically classified for elderly people, as in need of comprehensive assessment for risk factors. On the other hand, men more likely experienced overweight/obesity, but less likely had elevated waist circumference in comparison with women. This could be explained that men have greater abdominal visceral adipose tissue (likely corresponding to BMI), but less abdominal subcutaneous adipose tissue (likely corresponding to waist circumference) than women [77].

The study conducted on 153 hemodialysis patients in three dialysis centers in Tehran demonstrated that the prevalence of MetS among women was higher than that among men [62]. However, in the current study, gender was significantly associated with metabolic abnormalities, but not with AACE MetS or HMetS. This suggests that gender should take into consideration when assessing or treating patients with MetS, the presence of metabolic disorders in men or women may depend on their specific lifestyles and behaviors.

The longer hemodialysis vintage has shown the protective impact on MetS among studied hemodialysis patients. This somehow expressed the quality of hemodialysis in dialysis centers in Taiwan which reflected the effectiveness of multi-disciplinary care program in hospitals since 2003 to combat chronic kidney disease and related comorbidities [78]. In addition, the full reimbursement of dialysis costs by National Health Insurance in Taiwan medical system could further optimize the quality of care [79], which in turn reduced the prevalence of metabolic disorders in this study.

Physical activity was not associated with metabolic abnormalities or MetS in the present study. However, a review of several randomized trials concluded that the physical activity decreased the likelihood of development of MetS; if there were no contraindications, more intensive physical exercise or resistance training should be considered to prevent and treat MetS [63]. In addition, patients who performed regular exercise had better dialysis outcomes and health benefits as reported in an international study on hemodialysis patients [80].

Finally, the elevated level of hs-CRP did not show the association with MetS and its components. Inconsistently, the association was existed in the previous study, that inflammatory biomarkers had a correlation with MetS in hemodialysis patients [62]. An elevated level of hs-CRP may be a key independent predictor of adverse outcomes in hemodialysis patients with MetS. Therefore, reducing serum hs-CRP level should be considered for preventing MetS, CVD, and finally mortality among hemodialysis patients.

There was some limitations in the current study. Firstly, the causality cannot be proved between dietary EI and metabolic abnormalities and MetS in a cross-sectional design. In addition, the application of adequate EI but less MUFA intake was not clearly addressed because of the nature of the cross-sectional study design, and unavoidable reporting bias. More in-depth longitudinal studies and trials are required. The self-reported dietary assessment using food records and recalls had impacts on energy underreporting, appropriate interpretations of the results are recommended [81]. In the current study, we excluded those patients underreported their energy intake in order to avoid the bias and improve the reliability of findings [42]. However, the sample size is relatively small for subgroup analysis. Further investigation should be conducted on larger sample, to enhance the reliability of finding. The present study demonstrated a number of strengths that patients’ body composition was measured precisely and directly using the BIA, while biochemical data were assessed by using standard laboratory tests. Two MetS definitions reflecting the glucocentric, obesity, and CVD risk factors were used to assure the non-spuriousness of the relationships. Future longitudinal studies or trials were recommended to confirm the relationship between dietary intake and MetS and impacts of nutritional interventions on dialysis outcomes.

## Conclusions

This was the first study exploring the association of the reported dietary EI with metabolic abnormalities and MetS diagnosed by AACE and Harmonizing Metabolic Syndrome criteria in hemodialysis patients. We found that inadequate EI was high prevalence and associated with up to 2.26–8.17 folds of MetS and its components. Promoting adequate dietary energy intake following the K/DOQI guidelines could help to improve dialysis quality, prevent MetS, minimize the negative effects of metabolic disorders and their consequences, in turn, optimize the quality of care, and improve the quality of life of HD patients. Future studies are suggested for carefully exploring the mechanism, and evaluating the effect of dietary energy interventions.

## Abbreviations

AACE-MetS: Metabolic syndrome diagnosed by American Association of Clinical Endocrinologists
BIA: Bioelectrical impedance analysis
BMI: Body mass index
BP: Blood pressure
CCI: Charlson comorbidity index
CVD: Cardiovascular diseases
DBP: Diastolic blood pressure
EI: Energy intake
ESRD: End-stage renal disease
FPG: Fasting plasma glucose
HDL: High-density lipoprotein
HDL-C: High-density lipoprotein cholesterol
HMetS: Metabolic syndrome diagnosed by Harmonizing Metabolic Syndrome defined by Joint statement from the International Diabetes Federation (IDF), American Heart Association (AHA) and the National Heart, Lung, and Blood Institute (NHLBI), the World Heart Federation, the International Atherosclerosis Society, and the International Association for the Study of Obesity
hs-CRP: High sensitive C-reactive protein
IBW: Ideal body weight
IFG: Impaired fasting glucose
iPTH: Intact parathyroid hormone
LDL-C: Low-density lipoprotein cholesterol
MET: Metabolic equivalent minute/ week
MUFA: Mono-unsaturated fatty acid
NCEP- ATP III: National Cholesterol Education Program-Adult Treatment Panel-III definition
NKF-KDOQI: National Kidney Foundation-Kidney Disease Outcomes Quality Initiative
nPNA: Normalized protein nitrogen appearance
PUFA: Poly-unsaturated fatty acid
SBP: Systolic blood pressure;
SFA: Saturated fatty acid
TC: Total cholesterol
TG: Triglyceride
UFA: Unsaturated fatty acid
WC: Waist circumference

## References

[1] United States Renal Data System: International comparisons. The 2016 Annual data report: epidemiology of kidney disease in the United States: volume 2 – end-stage renal disease (ESRD) in the United States. In. USRDS Coordinating Center: National Institutes of Health, National Institute of Diabetes and Digestive and Kidney Diseases; 2016.

[2] Slee AD (2012). Exploring metabolic dysfunction in chronic kidney disease. Nutr Metab (Lond).

[3] Qian F, Korat AA, Malik V, Hu FB (2016). Metabolic effects of monounsaturated fatty acid–enriched diets compared with carbohydrate or polyunsaturated fatty acid–enriched diets in patients with type 2 diabetes: a systematic review and meta-analysis of randomized controlled trials. Diabetes Care.

[4] Kent PS, MP MC, Burrowes JD, Mc Cann L, Pavlinac J, Goeddeke-Merickel CM, Wiesen K, Kruger S, Byham-Gray L, Pace RC (2014). Academy of nutrition and dietetics and National Kidney Foundation: revised 2014 standards of practice and standards of professional performance for registered dietitian nutritionists (competent, proficient, and expert) in nephrology nutrition. J Acad Nutr Diet.

[5] Schoenaker DAJM, Mishra GD, Callaway LK, Soedamah-Muthu SS (2015). The role of energy, nutrients, foods, and dietary patterns in the development of gestational diabetes mellitus: a systematic review of observational studies. Diabetes Care.

[6] Beto JA, Ramirez WE, Bansal VK (2014). Medical nutrition therapy in adults with chronic kidney disease: integrating evidence and consensus into practice for the generalist registered dietitian nutritionist. J Acad Nutr Diet.

[7] Fouque D, Guebre-Egziabher F (2007). An update on nutrition in chronic kidney disease. Int Urol Nephrol.

[8] Kistler BM, Benner D, Burrowes JD, Campbell KL, Fouque D, Garibotto G, Kopple JD, Kovesdy CP, Rhee CM, Steiber A (2018). Eating during hemodialysis treatment: a consensus statement from the International Society of Renal Nutrition and Metabolism. J Ren Nutr.

[9] Kopple JD (2001). National Kidney Foundation K/DOQI clinical practice guidelines for nutrition in chronic renal failure. Am J Kidney Dis.

[10] Stark S, Snetselaar L, Hall B, Stone RA, Kim S, Piraino B, Sevick MA (2011). Nutritional intake in adult hemodialysis patients. Top Clin Nutr.

[11] St-Jules DE, Woolf K, Pompeii ML, Sevick MA (2016). Exploring problems in following the hemodialysis diet and their relation to energy and nutrient intakes: the BalanceWise study. J Ren Nutr.

[12] The National Kidney Foundation Kidney Disease Outcomes Quality Initiative (K/DOQI) Workgroup (2007). KDOQI clinical practice guidelines and clinical practice recommendations for diabetes and chronic kidney disease. Am J Kidney Dis.

[13] Kidney Disease Outcomes Quality Initiative (K/DOQI) Group (2003). K/DOQI clinical practice guidelines for management of dyslipidemias in patients with kidney disease. Am J Kidney Dis.

[14] The National Kidney Foundation Kidney Disease Outcomes Quality Initiative (K/DOQI) Workgroup. K/DOQI clinical practice guidelines on hypertension and antihypertensive agents in chronic kidney disease. Am J kidney dis. 2004;43. Supplement. 1:11–3. 15114537

[15] Tu S-F, Chou Y-C, Sun C-A, Hsueh S-C, Yang T (2012). The prevalence of metabolic syndrome and factors associated with quality of Dialysis among hemodialysis patients in southern Taiwan. Glob J Health Sci.

[16] Mottillo S, Filion KB, Genest J, Joseph L, Pilote L, Poirier P, Rinfret S, Schiffrin EL, Eisenberg MJ (2010). The metabolic syndrome and cardiovascular risk. A Systematic Review and Meta-Analysis J Am Coll Cardiol.

[17] Sattar N, McConnachie A, Shaper AG, Blauw GJ, Buckley BM, de Craen AJ, Ford I, Forouhi NG, Freeman DJ, Jukema JW (2008). Can metabolic syndrome usefully predict cardiovascular disease and diabetes? Outcome data from two prospective studies. Lancet.

[18] Kastorini CM, Panagiotakos DB, Georgousopoulou EN, Laskaris A, Skourlis N, Zana A, Chatzinikolaou C, Chrysohoou C, Puddu PE, Tousoulis D (2016). Metabolic syndrome and 10-year cardiovascular disease incidence: the ATTICA study. Nutr Metab Cardiovasc Dis.

[19] Harding J, Sooriyakumaran M, Anstey KJ, Adams R, Balkau B, Briffa T, Davis TME, Davis WA, Dobson A, Giles GG (2015). The metabolic syndrome and cancer: is the metabolic syndrome useful for predicting cancer risk above and beyond its individual components?. Diabetes Metab.

[20] Vogt BP, Souza PL, Minicucci MF, Martin LC, Barretti P, Caramori JT (2014). Metabolic syndrome criteria as predictors of insulin resistance, inflammation, and mortality in chronic hemodialysis patients. Metab Syndr Relat Disord.

[21] Einhorn D, Reaven GM, Cobin RH, Ford E, Ganda OP, Handelsman Y, Hellman R, Jellinger PS, Kendall D, Krauss RM (2003). American College of Endocrinology position statement on the insulin resistance syndrome. Endocr Pract.

[22] Alberti KGMM, Eckel RH, Grundy SM, Zimmet PZ, Cleeman JI, Donato KA, Fruchart J-C, James WPT, Loria CM, Smith SC (2009). Harmonizing the metabolic syndrome: a joint interim statement of the international diabetes federation task force on epidemiology and prevention; National Heart, Lung, and Blood Institute; American Heart Association; world heart federation; international atherosclerosis society; and International Association for the Study of obesity. Circulation.

[23] Martins Aline Moutinho, Dias Rodrigues Juliana Cordeiro, de Oliveira Santin Fernanda Galvão, Barbosa Brito Flavia dos Santos, Bello Moreira Annie Seixas, Lourenço Roberto Alves, Avesani Carla Maria (2015). Food Intake Assessment of Elderly Patients on Hemodialysis. Journal of Renal Nutrition.

[24] Shah A, Bross R, Shapiro BB, Morrison G, Kopple JD (2016). Dietary energy requirements in relatively healthy maintenance hemodialysis patients estimated from long-term metabolic studies. Am J Clin Nutr.

[25] Azadbakht L, Mirmiran P, Esmaillzadeh A, Azizi T, Azizi F (2005). Beneficial effects of a dietary approaches to stop hypertension eating plan on features of the metabolic syndrome. Diabetes Care.

[26] Daniel WW, Cross CL (2013). Biostatistics: A Foundation for analysis in the health sciences.

[27] Vogt BP, Ponce D, Caramori JCT (2016). Anthropometric indicators predict metabolic syndrome diagnosis in maintenance hemodialysis patients. Nutr Clin Pract.

[28] Pourhoseingholi MA, Vahedi M, Rahimzadeh M (2013). Sample size calculation in medical studies. Gastroenterol Hepatol Bed Bench.

[29] Wong T-C, Chen Y-T, Wu P-Y, Chen T-W, Chen H-H, Chen T-H, Hsu Y-H, Yang S-H (2016). Ratio of dietary ω-3 and ω-6 fatty acids—independent determinants of muscle mass—in hemodialysis patients with diabetes. Nutrition.

[30] Duong TV, Wong T-C, Chen H-H, Chen T-W, Chen T-H, Hsu Y-H, Peng S-J, Kuo K-L, Wang C-S, Tseng IH (2018). The cut-off values of dietary energy intake for determining metabolic syndrome in hemodialysis patients: a clinical cross-sectional study. PLoS One.

[31] Duong TV, Wong T-C, Su C-T, Chen H-H, Chen T-W, Chen T-H, Hsu Y-H, Peng S-J, Kuo K-L, Liu H-C (2018). Associations of dietary macronutrients and micronutrients with the traditional and nontraditional risk factors for cardiovascular disease among hemodialysis patients: a clinical cross-sectional study. Medicine (Baltimore).

[32] Wong T-C, Su H-Y, Chen Y-T, Wu P-Y, Chen H-H, Chen T-H, Hsu Y-H, Yang S-H (2016). Ratio of C-reactive protein to albumin predicts muscle mass in adult patients undergoing hemodialysis. PLoS One.

[33] Hemmelgarn BR, Manns BJ, Quan H, Ghali WA (2003). Adapting the Charlson comorbidity index for use in patients with ESRD. Am J Kidney Dis.

[34] Wong T-C, Chen Y-T, Wu P-Y, Chen T-W, Chen H-H, Chen T-H, Yang S-H (2015). Ratio of dietary n-6/n-3 polyunsaturated fatty acids independently related to muscle mass decline in hemodialysis patients. PLoS One.

[35] Okorodudu DO, Jumean MF, Montori VM, Romero-Corral A, Somers VK, Erwin PJ, Lopez-Jimenez F (2010). Diagnostic performance of body mass index to identify obesity as defined by body adiposity: a systematic review and meta-analysis. Int J Obes.

[36] Ipema KJR, Kuipers J, Westerhuis R, Gaillard CAJM, van der Schans CP, Krijnen WP, Franssen CFM (2016). Causes and consequences of Interdialytic weight gain. Kidney Blood Press Res.

[37] Liou YM, Jwo CJC, Yao KG, Chiang L-C, Huang L-H (2008). Selection of appropriate Chinese terms to represent intensity and types of physical activity terms for use in the Taiwan version of IPAQ. J Nurs Res.

[38] Craig CL, Marshall AL, Sjöström M, Bauman AE, Booth ML, Ainsworth BE, Pratt M, Ekelund U, Yngve A, Sallis JF (2003). International physical activity questionnaire: 12-country reliability and validity. Med Sci Sports Exerc.

[39] Lee PH, Macfarlane DJ, Lam TH, Stewart SM (2011). Validity of the international physical activity questionnaire short form (IPAQ-SF): a systematic review. Int J Behav Nutr Phys Act.

[40] Chiu Y-F, Chen Y-C, Wu P-Y, Shih C-K, Chen H-H, Chen H-H, Chen T-H, Yang S-H (2014). Association between the hemodialysis eating index and risk factors of cardiovascular disease in hemodialysis patients. J Ren Nutr.

[41] Shapiro BB, Bross R, Morrison G, Zadeh K, Kopple JD (2015). Self-reported, interview-assisted diet records underreport energy intake in maintenance hemodialysis patients. J Ren Nutr.

[42] Hirvonen T, Männistö S, Roos E, Pietinen P (1997). Increasing prevalence of underreporting does not necessarily distort dietary surveys. Eur J Clin Nutr.

[43] McDoniel SO (2007). Systematic review on use of a handheld indirect calorimeter to assess energy needs in adults and children. Int J Sport Nutr Exerc Metab.

[44] Nieman DC, Trone GA, Austin MD (2003). A new handheld device for measuring resting metabolic rate and oxygen consumption. J Am Diet Assoc.

[45] St-Onge M-P, Rubiano F, Jones A, Heymsfield SB (2004). A new hand-held indirect calorimeter to measure postprandial energy expenditure. Obes Res.

[46] Hasson RE, Howe CA, Jones BL, Freedson PS (2011). Accuracy of four resting metabolic rate prediction equations: effects of sex, body mass index, age, and race/ethnicity. J Sci Med Sport.

[47] Wu P-Y, Yang S-H, Wong T-C, Chen T-W, Chen H-H, Chen T-H, Chen Y-T (2015). Association of Processed Meat Intake with hypertension risk in hemodialysis patients: a cross-sectional study. PLoS One.

[48] Daugirdas JT (1995). Simplified equations for monitoring Kt/V, PCRn, eKt/V, and ePCRn. Adv Ren Replace Ther.

[49] Shaw JE, Zimmet PZ, Alberti KGMM (2006). Point: impaired fasting glucose: the case for the new American Diabetes Association criterion. Diabetes Care.

[50] Hwang L-C, Bai C-H, Chen C-J (2006). Prevalence of obesity and metabolic syndrome in Taiwan. J Formos Med Assoc.

[51] Expert Panel on Detection Evaluation and Treatment of High Blood Cholesterol in Adults (2001). Executive summary of the third report of the national cholesterol education program (NCEP) expert panel on detection, evaluation, and treatment of high blood cholesterol in adults (adult treatment panel III). JAMA.

[52] Omae Kenji, Kondo Tsunenori, Tanabe Kazunari (2015). High preoperative C-reactive protein values predict poor survival in patients on chronic hemodialysis undergoing nephrectomy for renal cancer. Urologic Oncology: Seminars and Original Investigations.

[53] McAuley KA, Williams SM, Mann JI, Walker RJ, Lewis-Barned NJ, Temple LA, Duncan AW (2001). Diagnosing insulin resistance in the general population. Diabetes Care.

[54] Ascaso JF, Pardo S, Real JT, Lorente RI, Priego A, Carmena R (2003). Diagnosing insulin resistance by simple quantitative methods in subjects with normal glucose metabolism. Diabetes Care.

[55] Kidney Disease (2009). Improving global outcomes (KDIGO) CKD–MBD work group. KDIGO clinical practice guideline for the diagnosis, evaluation, prevention, and treatment of chronic kidney disease–mineral and bone disorder (CKD–MBD). Kidney Int.

[56] Lopes AA, Bragg-Gresham JL, Elder SJ, Ginsberg N, Goodkin DA, Pifer T, Lameire N, Marshall MR, Asano Y, Akizawa T (2010). Independent and joint associations of nutritional status indicators with mortality risk among chronic hemodialysis patients in the Dialysis outcomes and practice patterns study (DOPPS). J Ren Nutr.

[57] Shapiro SS, Wilk MB (1965). An analysis of variance test for normality (complete samples). Biometrika.

[58] Razali NM, Wah YB (2011). Power comparisons of shapiro-wilk, kolmogorov-smirnov, lilliefors and Anderson-darling tests. Journal of Statistical Modeling and Analytics.

[59] Sicras-Mainar A, Ruíz-Beato E, Navarro-Artieda R, Maurino J (2017). Comorbidity and metabolic syndrome in patients with multiple sclerosis from Asturias and Catalonia, Spain. BMC Neurol.

[60] Hildrum B, Mykletun A, Hole T, Midthjell K, Dahl AA (2007). Age-specific prevalence of the metabolic syndrome defined by the international diabetes federation and the National Cholesterol Education Program: the Norwegian HUNT 2 study. BMC Public Health.

[61] Razzouk L, Muntner P (2009). Ethnic, gender, and age-related differences in patients with the metabolic syndrome. Curr Hypertens Rep.

[62] Shahrokh S, Heydarian P, Ahmadi F, Saddadi F, Razeghi E (2012). Association of Inflammatory Biomarkers with metabolic syndrome in hemodialysis patients. Ren Fail.

[63] Lakka TA, Laaksonen DE (2007). Physical activity in prevention and treatment of the metabolic syndrome. Appl Physiol Nutr Metab.

[64] Cuppari L, Ikizler TA (2010). Energy balance in advanced chronic kidney disease and end-stage renal disease. Semin Dial.

[65] Beto JA, Bansal VK (2004). Medical nutrition therapy in chronic kidney failure: integrating clinical practice guidelines. J Am Diet Assoc.

[66] Veeneman JM, Kingma HA, Boer TS, Stellaard F, De Jong PE, Reijngoud D-J, Huisman RM (2003). Protein intake during hemodialysis maintains a positive whole body protein balance in chronic hemodialysis patients. Am J Physiol Endocrinol Metab.

[67] Young DO, Lund RJ, Haynatzki G, Dunlay RW (2007). Prevalence of the metabolic syndrome in an incident dialysis population. Hemodial Int.

[68] Um Y-J, Oh S-W, Lee C-M, Kwon H-T, Joh H-K, Kim Y-J, Kim H-J, Ahn S-H (2015). Dietary fat intake and the risk of metabolic syndrome in Korean adults. Korean J Fam Med.

[69] Gillingham LG, Harris-Janz S, Jones PJH (2011). Dietary monounsaturated fatty acids are protective against metabolic syndrome and cardiovascular disease risk factors. Lipids.

[70] Riccardi G, Giacco R, Rivellese AA (2004). Dietary fat, insulin sensitivity and the metabolic syndrome. Clin Nutr.

[71] Esposito K, Maiorino MI, Ceriello A, Giugliano D (2010). Prevention and control of type 2 diabetes by Mediterranean diet: a systematic review. Diabetes Res Clin Pract.

[72] Koloverou E, Esposito K, Giugliano D, Panagiotakos D (2014). The effect of Mediterranean diet on the development of type 2 diabetes mellitus: a meta-analysis of 10 prospective studies and 136,846 participants. Metabolism.

[73] Martínez-González MÁ, Martín-Calvo N (2013). The major European dietary patterns and metabolic syndrome. Rev Endocr Metab Disord.

[74] Calton EK, James AP, Pannu PK, Soares MJ (2014). Certain dietary patterns are beneficial for the metabolic syndrome: reviewing the evidence. Nutr Res.

[75] Kastorini C-M, Milionis HJ, Esposito K, Giugliano D, Goudevenos JA, Panagiotakos DB (2011). The Effect of Mediterranean Diet on Metabolic Syndrome and its Components: A Meta-Analysis of 50 Studies and 534,906 Individuals. J Am Coll Cardiol.

[76] Buscemi S, Verga S, Donatelli M, D’Orio L, Mattina A, Tranchina MR, Pizzo G, Mulè G, Cerasola G (2009). A low reported energy intake is associated with metabolic syndrome. J Endocrinol Investig.

[77] Maki KC, Rains TM, Bell M, Reeves MS, Farmer MV, Yasunaga K (2011). Fat mass, abdominal fat distribution, and C-reactive protein concentrations in overweight and obese men and women. Metab Syndr Relat Disord.

[78] Chen Y-R, Yang Y, Wang S-C, Chiu P-F, Chou W-Y, Lin C-Y, Chang J-M, Chen T-W, Ferng S-H, Lin C-L (2013). Effectiveness of multidisciplinary care for chronic kidney disease in Taiwan: a 3-year prospective cohort study. Nephrol Dial Transplant.

[79] Cheng T-M (2015). Reflections on the 20th anniversary of Taiwan’s single-payer National Health Insurance System. Health Aff.

[80] Tentori F, Elder SJ, Thumma J, Pisoni RL, Bommer J, Fissell RB, Fukuhara S, Jadoul M, Keen ML, Saran R (2010). Physical exercise among participants in the Dialysis outcomes and practice patterns study (DOPPS): correlates and associated outcomes. Nephrol Dial Transplant.

[81] Subar AF, Freedman LS, Tooze JA, Kirkpatrick SI, Boushey C, Neuhouser ML, Thompson FE, Potischman N, Guenther PM, Tarasuk V (2015). Addressing current criticism regarding the value of self-report dietary data. J Nutr.

